# Supplementary material for: Subregion and sex differences in ethanol activation of cholinergic and glutamatergic cells in the mesopontine tegmentum
Source: Sci Rep. 2024 Jan 2;14:46. doi: 10.1038/s41598-023-50526-1 (PMC10762073; doi:10.1038/s41598-023-50526-1)
Supplement: Supplementary file 1 — Supplementary Figures. [file 41598_2023_50526_MOESM1_ESM.docx]

**Supplementary Material**


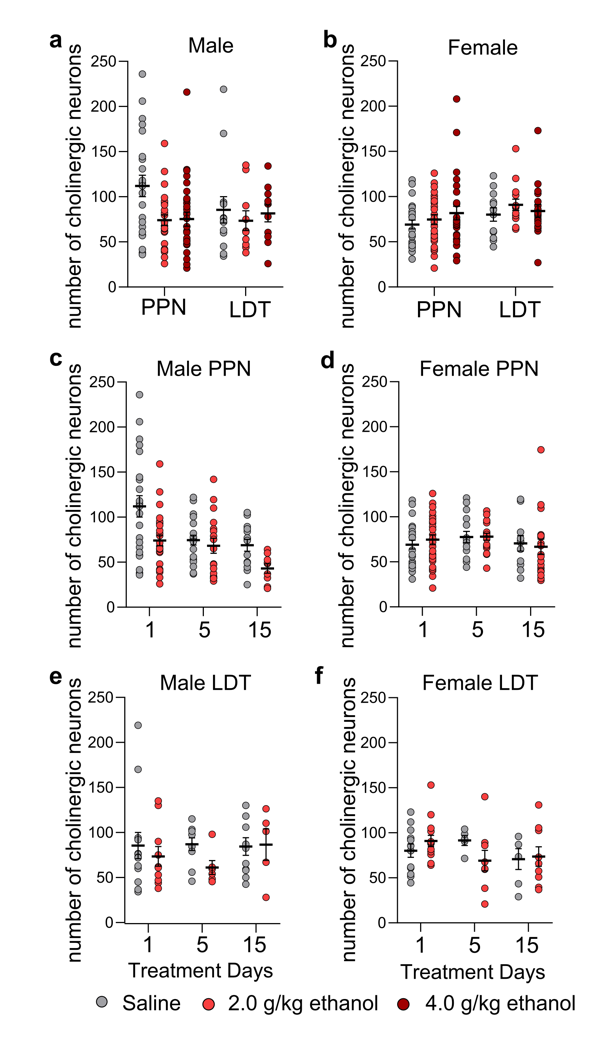


Figure S1. **The number of cholinergic neurons identified by IHC in the MPT is not different across sex, MPT subregion or treatment group.** The number of cholinergic neurons in **a)** male PPN and LDT, and **b)** female PPN and LDT acutely treated with saline-, 2 or 4 g/kg ethanol. The number of cholinergic neurons in **c)** male PPN, **d)** female PPN, **e)** male LDT and **f)** female LDT chronically treated with 1, 5 or 15 daily injections of saline or 2 g/kg ethanol. The number of ROI and animals per group are listed in figure legend 2. Data expressed as mean ± SEM.


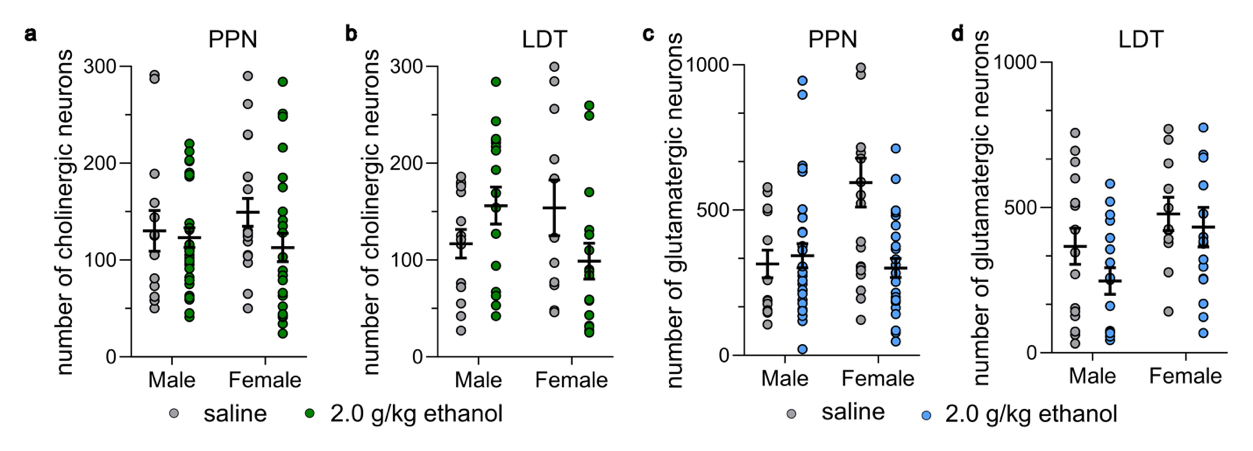


Figure S2. **The number of cholinergic and glutamatergic neurons identified by RNAscope in the MPT is not different across sex, MPT subregion or treatment group.** The number of cholinergic neurons in the **a)** PPN and **b)** LDT and the number of glutamatergic neurons in the **c)** PPN and **d)** LDT in male and female mice chronically treated with 15 daily injections of saline- or 2 g/kg ethanol. The number of ROI and animals per group are listed in figure legends 4 and 5. Data expressed as mean ± SEM.

w
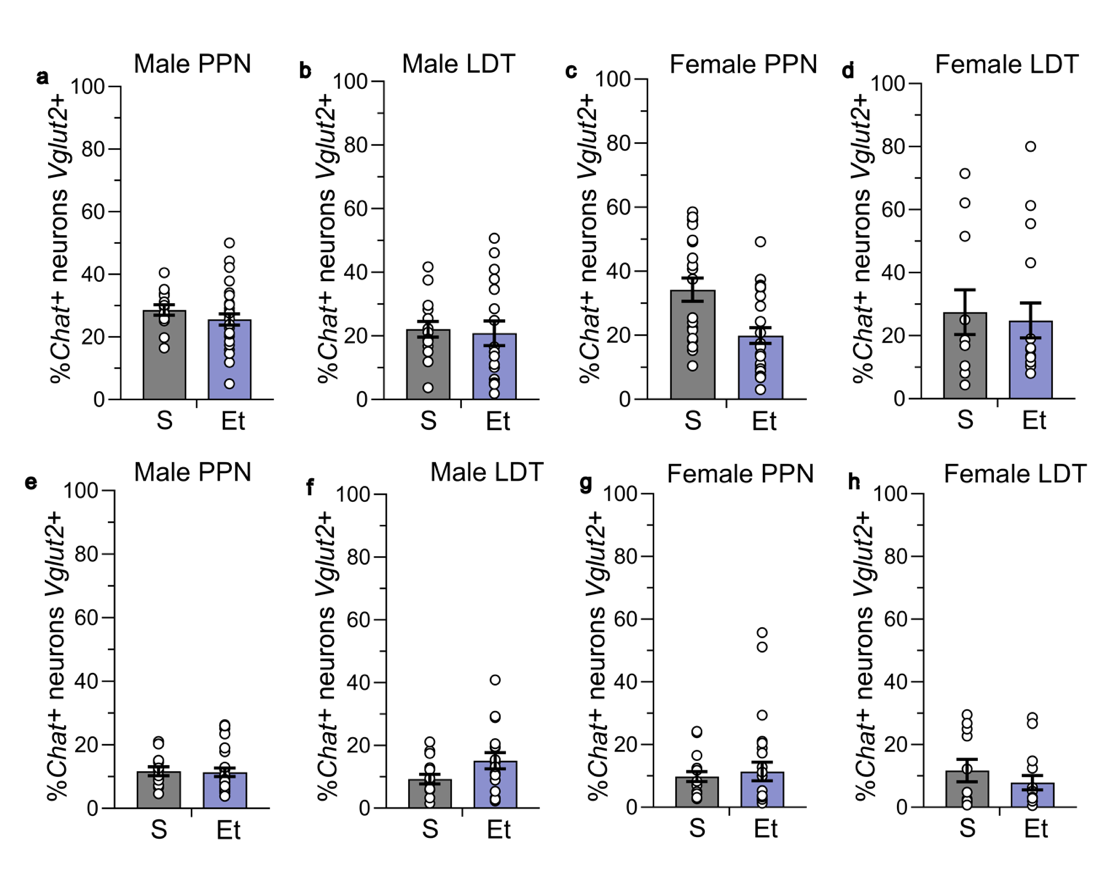


Figure S3. **The number of co-labeled cholinergic and glutamatergic neurons identified by RNAscope in the MPT is not different across sex, MPT subregion or treatment group.** The percent of *Chat*-positive neurons in the *Vglut2*-positive population is similar between the saline and chronic-ethanol treated groups in the male **a)** PPN and **b)** LDT and female **c)** PPN and **d)** LDT. The percent of *Vglut2*-positive neurons in the *Chat*-positive population is similar between groups in the male **e)** PPN and **f)** LDT and female **g)** PPN and **h)** LDT. The number of ROI and animals per group are listed in figure legends 4 and 5. Data expressed as mean ± SEM.
